# Burning Issues: Cremation and Incineration in Modern India

**DOI:** 10.1007/s00048-017-0158-7

**Published:** 2017-02-24

**Authors:** David Arnold

**Affiliations:** Department of History, University of Warwick, CV4 7AL Coventry, UK

**Keywords:** Cremation, Incineration, Environment, Infrastructure, Feuerbestattung, Müllverbrennung, Umwelt, Infrastruktur

## Abstract

The cremation of human bodies and the incineration of urban waste provide two interrelated examples of technologies using the destructive power of fire that “travelled” in both directions between India and the West in the nineteenth and twentieth centuries. Rather than granting an automatic ascendency to western ways of burning the dead or disposing of urban rubbish, these case studies indicate the manner in which culture and environment inhibited or prevented their advance and favoured the survival or re-articulation of pre-existing technological practices and the socio-political infrastructure in which they were embedded. In the process of travelling, in part made possible by the agency of colonial personnel and the instruments of imperial exchange, but also through Indian opinion and diasporic dissemination, some technologies substantially changed their meaning, context and material form while others, seemingly untouched, underwent more subtle transformation.

## Zusammenfassung

Zu den zahlreichen Techniken, die im 19. und 20. Jahrhundert zwischen Indien und dem Westen zirkulierten, gehörten die Einäscherung menschlicher Leichen sowie die Verbrennung städtischer Abfälle, die in enger Wechselbeziehung zueinander standen. Dieser Beitrag hinterfragt die Auffassung, dass sich westliche Verbrennungstechniken und Praktiken zur Bestattung und Müllentsorgung global durchsetzten. Mit seinen beiden Fallstudien zeigt der Autor vielmehr, wie Kultur- und Umweltfaktoren den technischen Wandel hemmten oder unterbanden und stattdessen den Fortbestand bzw. die Modifikation implementierter technischer Verfahren begünstigten. Im Laufe der Austauschprozesse, die durch die Politik und Instrumente kolonialer Verwaltung genauso wie durch die öffentliche Meinung in der Diaspora ausgelöst wurden, veränderten einige Techniken ihre Bedeutung, ihre Rahmenbedingungen und ihre konkrete materielle Ausformung – andere blieben von diesen Prozessen unberührt oder wandelten sich auf subtile Weise.

In the age of empire questions of technology have frequently been presented in terms that pre-emptively favour Western science and innovation. In the nineteenth and early twentieth centuries, such exemplary technologies as modern weaponry, steam power, electricity, railways and telegraphs were self-evidently products of the West at a dynamic stage of its industrial and scientific evolution. Their introduction and dissemination in the non-West, a process conventionally termed “technology transfer”, was bound to create a sense of disparity that privileged Europe and North America over contemporary Asia, Africa and Latin America (Headrick 1981). There are, however, ways of qualifying or redressing this Eurocentrism. One way is to consider technologies as actively travelling rather than being passively transferred. The question then becomes: how are technologies transformed as they move between (and within) different societies, as they travel through space and time? In some instances, the technological skills and artefacts of the machine age were resisted and rejected: they failed to travel at all. But others were selectively redeployed, given new meanings and contexts, and so, through local incorporation, adaptation and innovation, assumed a socio-cultural identity, and even a physical form, substantially different from that originally intended. As elements in a new environment and a new infrastructure, they became reconstituted through need, use and experience.

The controlled use of fire was one of the most ancient and elementary technologies known to humans: the focus here is, however, on fire as an imperfect instrument of purification and destruction. I use the interrelated processes involved in human cremation and urban waste incineration to further an argument about the need for a “more interactive, culturally-nuanced, multi-sited” discussion of technology in the non-Western world and its functioning within “specific parameters of time, place and culture” (Arnold 2005: 85). Using English-language texts, sanitary tracts, municipal records and newspaper reports from the 1860s onwards, this article examines how the use of fire to consume bodies and destroy urban waste evolved alongside each other in the cities of modern India. Here the environment represents both resource and effect, especially since both cremation and incineration were subsets of a wider issue of urban smoke pollution and its regulation.^1^ Infrastructure signifies both the physical elements (roads, railways, crematoria, incinerators) around and through which the modern city functioned and the assemblage of socio-political values and practices (religion, caste, nationhood) that fashioned or critiqued technological goods and processes.

Both cremation and incineration were primarily urban issues, forming a highly visible and contentious part of Indian urban infrastructure by the late nineteenth century. Given their size and their prominence as centres of colonial administration and European residence, of Indian social life and commercial activity, the cities of British India and their municipal corporations constituted exceptional sites of observation and scrutiny, of environmental regulation and public protest. Cremation and incineration might not normally be considered “everyday technologies” (Arnold 2013a), and yet they contained significant elements of the familiar and quotidian. This was apparent in the conspicuousness of urban cremation grounds, in the routine collection and destruction of urban waste, in the citywide impact of smoke from funeral pyres and incineration plants, and in the elemental and accessible processes involved in destruction by fire. This familiarity—repeatedly made manifest to the senses through sight and smell—helped locate the technology of the crematorium and the incinerator within the domain of public concern and not merely that of state policy and “expert” opinion.

But while there were many similarities between these technologies, there were also salient differences. Contrary to diffusionist narratives, and as a long established means for the disposal of the dead and a prestigious religious rite, especially among upper-caste Hindus, the cremation of human remains in India was only marginally modified by the 1870s rise of the modern cremation movement in the West (a movement India helped inspire) and the industrial technology it employed. The Indian practice of wood-fuelled, open-air cremation remained not only defiantly opposed to Western innovation but was also formally incorporated into the evolving urban infrastructure and ultimately recast as an emblem of national identity. By contrast, removing and burning urban waste had none of the positive cultural connotations attached to human cremation among Hindus and some other religious communities in India. However, attempts to follow the British precedent of installing large incinerators (called “destructors”) to dispose of urban waste met with only chequered success. This was partly due to high installation and maintenance costs and unacceptable levels of air pollution (considerations that had Western parallels), but also because the Indian environment, the physical nature of Indian waste and established waste-disposal practices militated against the wholesale adoption of this technological innovation. In the cremation case, ideas about the desirability of burning as a means of disposing of the dead travelled in both directions, to and from Europe, and received further impetus from the South Asian diaspora: even then Indian and Western practices remained distinct. With incineration, the traffic in technological objects and expertise was essentially one-way—from Britain to India—but it gained no automatic acceptance thereby. Indeed, just as “modern” means of waste-disposal took on many “traditional” characteristics in India, so a degree of local innovation was involved in trying to make incineration practicable in India. In neither case is it sufficient simple to write in “Europe” or even “Empire” when the internal and interregional traffic in technology was far more complex, multi-sited and locally entangled.

## Cremation: Perception and Practice

Burning the bodies of the dead was an ancient rite and practice in India. It was observed among Buddhists, Hindus and Jains from well before the start of the Common Era, and was later adopted by Sikhs. Although not all Hindus practised cremation (most lower-caste Hindus were buried), burning the dead historically helped demarcate these religious communities from Muslims and Christians, for whom burial was the norm, and from India’s Parsi community who exposed their dead on Towers of Silence. Although the rituals accompanying cremation varied between different communities (and within them), there was a shared belief that cremation should take place as soon as possible after death, usually within 24 hours, in the open air and on a pyre made of wood (Parry 1994; Rambachan 2003). While the primary rationale was religious—to free the soul from the defunct body—and grounded in sacred text and ancient custom, sanitary arguments were sometimes made for cremation, especially the rapid decomposition caused by a hot, humid climate. Some nineteenth-century writers claimed that cremation originated in India, with the “Aryans” or their non-“Aryan” predecessors. Originating at a time when India was still heavily forested, cremation may also have been environmentally more appropriate and sustainable than, for instance, the mummification practised in the dry desert air of ancient Egypt. Cremation was thought to have spread from South Asia to other parts of Eurasia, thereby constituting an early form of technological and cultural diffusion (Eassie 1875: 4; Erichsen 1887: 7; Richardson 1893: 1–2, 11). Whatever the origins of the practice, Europeans in the imperial age were very much aware that cremation was far more widely practised in India than anywhere else, where it was often said (misleadingly) to be “all but universal” among Hindus (Eassie 1875: 35). The identification of cremation with ancient Hindu and Buddhist civilization (as well as with Greece and Rome) was even more marked in North America (Prothero 2001: 19, 39, 81). However, recognition of cremation’s historical roots in India and contemporary evidence of its widespread observance there did not necessarily create a favourable impression of the actual nature of Indian practice among western commentators. The idea of cremation travelled more readily from East to West than the actual technology of its performance.

Until the 1870s cremation was widely regarded in the West as inhumane and abhorrent, un-Christian, and evidence of Hindus’ “heathen” ways and “barbaric” customs. It is possible that the rite of *sati*, or self-immolation, by which a Hindu widow was burned on the funeral pyre of her husband, added to the sense of horror and repugnance that cremation engendered. Despite the outlawing of *sati* by the English East India Company in 1829, many negative associations remained attached to cremation, especially among Europeans resident in India. Indeed, as direct Western involvement in India increased from the mid-eighteenth century onwards, it was common for Europeans to voice intense disgust at the sight and smell of burning corpses on riverbanks and in cremation grounds (Fay 1925: 207–208; Lawrence & Woodiwiss 1980: 158–159). Apart from personal sensibilities, Europeans decried cremation as a health hazard due to the clouds of foul smoke issuing from the burning grounds or *ghat*s. In 1859, Bengal’s inspector-general of jails complained about smoke from a nearby Hindu cremation site drifting into the prison at Hooghly. The smell was “nauseous and disgusting to the last degree”, he protested, and on some days so repulsive that the prisoners could not bear to eat their food.^2^ As we will see shortly, similar complaints were made about the burning of urban waste.

Before the 1860s Hindu cremation was unregulated by state agency, occurring in places, usually close to a river or by the sea, where the ashes could be immersed in water and preferably in sacred rivers, such as the Ganges. Although the burning of the dead was attended by relatives and friends of the diseased, as well as by priests and the attendants who provided wood and other necessities, the sites themselves were unpoliced and unbounded. Worse still, in the Western view, was the way how half-charred bones and unburnt body-parts littered riverbanks and streams as a result of incomplete incineration, attracting dogs, jackals and vultures. Fire could be a very imperfect means of disposing of human remains—just as it proved to be for urban waste. During famines and epidemics, when firewood was scarce and the number of dead immense, or even in more normal times when the costs of cremation were too high for the poor to afford, bodies might simply be dumped in rivers or abandoned on their banks (Bengal 1865: 80). Hence, far from constituting a favourable model, worthy of emulation, to most Western eyes cremation in India presented a practice that was technically deficient as well as morally repugnant. European disgust at Indian burial and burning grounds became instead an incentive for early schemes to construct in-door crematoria in India (Martin 1864: 1–2). For compelling social and political reasons, there could be no question of the colonial government banning cremation, but ways had to be found within the evolving urban infrastructure to accommodate it and make it, as far as possible, compliant with sanitary expectations and environmental controls. From the mid-nineteenth century, as East India Company rule was replaced by the British Crown, India’s provincial governments and newly constituted municipal authorities sought to remove Hindu cremation grounds from the centres of Calcutta (Kolkata) and Bombay (Mumbai) and either to relocate them to the urban periphery or reduce the “nuisance” of those that remained. Some officials called for high perimeter walls to restrict the public visibility of cremation grounds or proposed that an adequate quantity of wood be available to ensure the complete destruction of human remains. Prominently marked on maps along with Christian cemeteries and Muslim burial grounds, cremation grounds became conspicuous features of the urban landscape: they were also one of the sites through which the municipal authorities sought to enumerate the dead and register causes of mortality (Bombay 1867: 6; Conybeare 1855, appendix H, 16; Buckland 1901: 1, 280–282, 296–297).

Hindu cremation commanded its own social infrastructure from the Brahmin priests, who presided over funeral rites, down to the low-caste attendants, who haggled with mourners over the price of wood (Madras 1922: 31). The burning of the dead required large quantities of fuel, including, among opulent Hindus, expensive sandalwood and copious amounts of ghee and coconut oil to fuel the flames and disguise the smell of burning flesh. By contrast, the cremations of Indian paupers in early twentieth-century Shanghai were cheap and simple—a dozen bundles of firewood, a cotton winding sheet and some oil, costing altogether no more than ten to twenty dollars.^3^ Birendra Nath Ghosh calculated that to burn an adult body and reduce it to ashes in three hours required 400 pounds of wood at a cost of roughly six rupees (equivalent at the time to 10 shillings). Apart from sandalwood, he identified the “beautiful” wood felled from trees that grew along the Bengal delta and in the Sundarbans forest (Ghosh 1930: 339): other types of wood were favoured elsewhere in India. If we assume that each cremation required 400 pounds of fuel, then 4,000 tons of wood would have been needed in Bombay in 1897 for the 22,818 cremations held in that year and 2,800 tons in Calcutta in 1917/18 when 15,489 corpses were cremated (Bombay 1898: 64; Calcutta 1918: 1, 86)—allowing that some cremations used less others more wood than this. Cremation on such a scale thus required a large and constant supply of wood—one reason why there was a large number of wood-yards in Indian cities. For instance, in Bombay in 1882 the municipal authorities issued 1823 licenses for timber-yards, including 195 that sold sandalwood (Bombay 1883: 370). The use of wood in cremation pyres created additional pressure for legal (and illegal) timber extraction from state-managed forests and private woodlands.^4^ It was often argued in the West that cremation was, as we would now put it, “environmentally friendly”—needing less space than graveyards, cremation freed land for more productive uses—but in India cremation was a fuel-hungry, polluting technology that bore considerable environmental costs.^5^

In the late nineteenth century a significant shift occurred in attitudes to cremation in both India and the West. One factor in this was the rise of cremation societies—in Italy, France, Germany, Britain and the United States—which campaigned for the legalization of cremation and the building of crematoria for the indoor burning of the dead (Parsons 2003: 611–612; Jupp 2006: 46–69). Apart from land shortages in cities like London, the cremation movement in the West was also driven by a growing secularization of attitudes towards the dead. Further, with its high-temperature ovens and factory-like chimneys, the crematorium represented an extension to funeral practices of the technological capacities of an industrial age, of blast furnaces and steel making: cremation became the “industrial annihilation of bodies” (Laqueur 2015: 510). India, still early in its industrialization, was ill-equipped to match these technological advances and showed little evidence of secularization. However, given the scale and antiquity of cremation in South Asia, India served the movement as a model, in which the less desirable aspects of local practice were ignored or minimized, and as an authoritative demonstration that cremation was not, as critics claimed, inhumane and godless. Physicians and administrators with Indian experience became de facto experts, qualifying claims about the supposed universality of cremation in India while plying European audiences with accounts of how well and how nobly cremation was managed in India (Robinson 1889: 186–188). William Eassie, a leading figure in the Cremation Society of England, cited contemporary as well as ancient India to show that the cremation was “neither new in theory nor in practice” (Eassie 1875: 1). Guided by press reports and personal correspondence, he referred in particular to the recent cremation of Narayan Wasudeo, a prominent citizen of Bombay in 1874 and that of the Maharaja of Kolhapur, who died in Florence in November 1870 and whose body was ceremonially burned in a public park alongside the Arno (Eassie 1875: 90, 94). The technical details of the funeral pyre, the time needed for the body to be consumed, the rituals performed, and the “enlightened” attitude of the Florentine authorities—all attracted comment at a time when the morality and legality of cremation were still hotly debated in Europe.

As will be seen shortly, the needs of Hindus and Sikhs who died abroad were also a significant factor in the spread of cremation outside India. Among the first thirty individuals cremated at the Woking Crematorium in Surrey, following its inauguration in 1885, were a Brahmin woman and a follower of the Hindu reform organization, the Arya Samaj (*Times of India*, 4 March 1887: 6; Thompson 1899: 24). Although Indians were among the beneficiaries of this first working crematorium in Britain, they had played no direct part in its creation.^6^ Thirty years later, when neglect of their customary funeral practices stirred discontent among Indian soldiers sent to the Western Front, the British military authorities responded by permitting their cremation. The bodies of 53 Hindu and Sikh soldiers, previously hospitalized in Brighton, were burned on the Downs above the town, one of only two occasions when open-air cremation was permitted in Britain (White 2005).

In British India, too, support for cremation was growing, though not necessarily along lines favoured in the West. Not only did Hindus strenuously defend their practice against European criticism (*Correspondence* 1855: 13–14; Ghosh 1930: 339); the colonial authorities themselves began to regard it with qualified approval. For them the principal argument in favour of cremation was not religious but sanitary. Invoking contemporary western ideas of miasmatic poison and epidemic contagion, colonial medical officers argued that Indian burial grounds had become dangerously overcrowded and unhealthy. Compared to European burial grounds, they were breeding grounds for disease (Douglas 1867: 24–31; Hehir 1913: 408–411). Cremation, by contrast, when properly conducted, was deemed a safer, more sanitary way of disposing of bodies and of eliminating the miasmas emanating from decomposing corpses (Bombay 1873: 15). This implied, however, a reform of existing practices with tight restrictions on open-air cremation sites or the construction, along Western lines, of enclosed, brick-built crematoria. From the 1880s, the *Indian Medical Gazette*, India’s premier medical journal, campaigned for “scientific” cremation, arguing that, as the custom of “a large majority of the population”, cremation already enjoyed far greater approval in India than it did in the West (Editorial 1887: 175). In February 1894, the *Gazette* argued that from a sanitary and environmental perspective cremation was by far the best means of disposing of human bodies in conditions where putrefaction was rapid and the risk immense of transmitting typhoid, cholera and other diseases from the dead to the living. If an infected body was buried, the “germs of disease” would eventually infect the soil, groundwater and air. The only effective way to prevent this was through the “destruction of disease germs by heat”. This “speedy, effectual and easy method” not only conveniently disposed of the dead but also ensured that their remains inflicted “the minimum of harm upon the living” (Editorial 1894a: 60). By the 1920s, authoritative texts like *McNally’s Sanitary Handbook* stated that “cremation is the most sanitary method of disposal of the dead […]. When properly carried out, the method is without objection” (Russell 1923: 247).

## Cremation Nation

Among Indians, too, cremation was acquiring a growing popularity. In the past cremation had largely been the preserve of high-status Hindus, with members of the lower castes, unless exceptionally wealthy, burying their dead. Infants of all castes were also buried. Cremation was not, therefore, the “universal” practice among Hindus it was often presumed to be. For instance, in Bombay in 1880 only 43 percent of the Hindus who died in the city were cremated—that is, 5,569 out of the 13,037 dead (Bombay 1881: 320).^7^ This distinction was, however, beginning to erode as more Hindus, regardless of caste, chose to follow the prestigious rite of open-air cremation or aspired to it as part of their social reform agenda.^8^ Behind this shift one can see an extension to death practices of what has been termed “sankritization” as lower castes adopted the ritual practices and social privileges of the higher castes. There is, however, little clear evidence for this change before the 1890s. In Bombay between 1873 and 1893, and excluding Parsis whose bodies were exposed on the Towers of Silence, cremation rose only slightly from about 30 to around 33 percent of those who died in the city. However, in 1897, as bubonic plague struck the city, causing 11,000 deaths, 24,812 corpses were buried and 22,818 burned, temporally raising the proportion of those cremated to nearly 48 percent (Bombay 1898: 64).

**Fig. 1:**
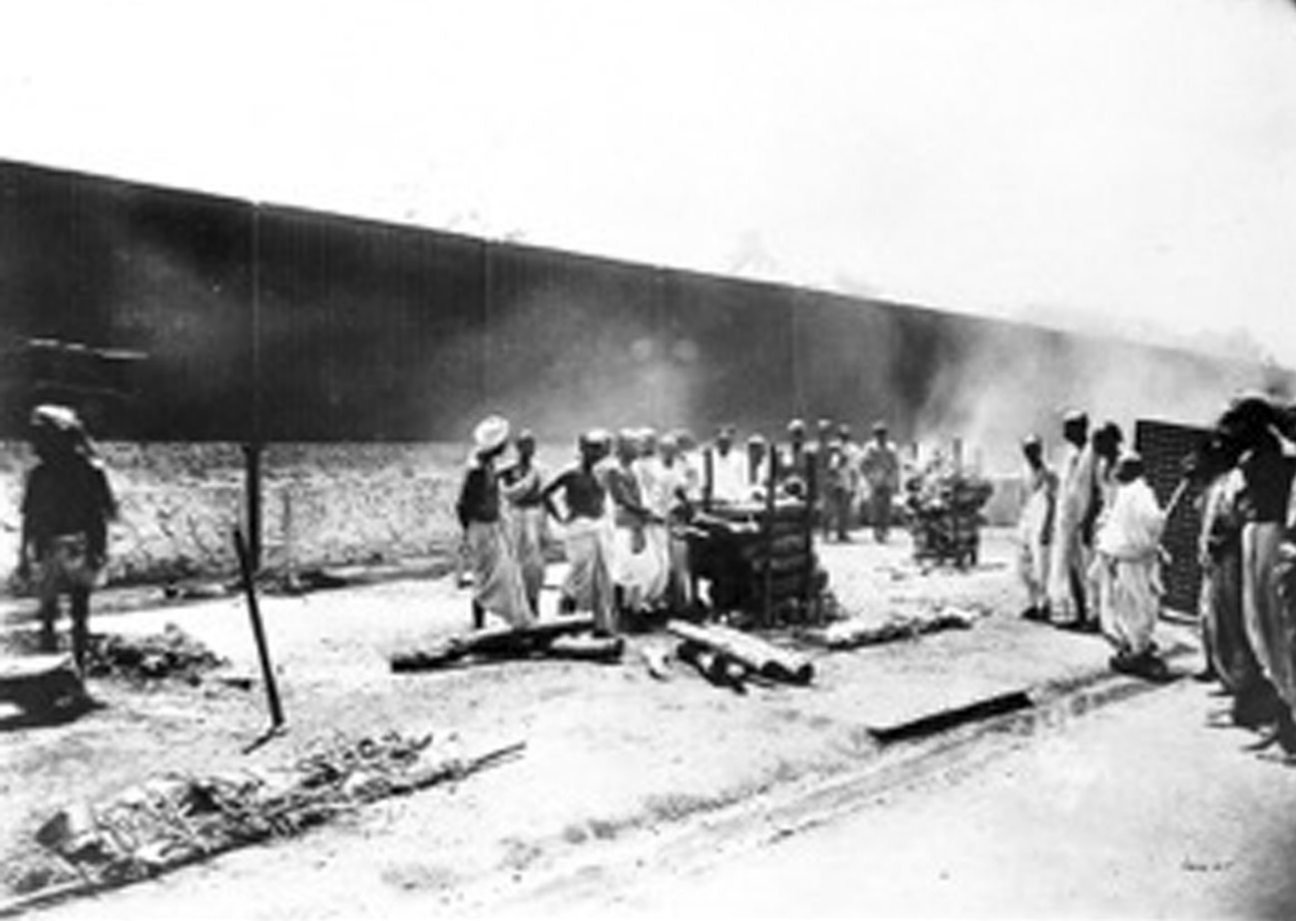
Cremation in Bombay during the Plague Epidemic, 1897

In addition to a long-term shift towards cremation among Hindus, a further factor in its increase was the growing reliance of municipal authorities on cremation to dispose of the unclaimed dead, especially during famines and epidemics. It is partly this municipal recourse to cremation that explains the sharp rise in Bombay in 1897. Earlier, in 1883, in the wake of a famine in which thousands of sick and impoverished rural migrants entered the city and many died on its streets, Bombay municipality sanctioned 30,000 rupees a year for the cremation of “the poor dead of the Hindu community” (*Times of India*, 22 February 1888: 3). Sixty years later, during the Bengal famine of 1943, as the famine poor drifted into Calcutta and many perished there, a police disposal squad was charged with collecting dead bodies and transporting them to the city’s burning grounds and crematoria (*Times of India*, 27 September 1943: 6). This use of cremation to dispose of the bodies of destitute, mostly low-caste migrants, and the practical value of using Western-style crematoria for the purpose, sparked intense debates among the Indian middle class about the merits (or otherwise) of “scientific” cremation. In Bombay, in the 1880s and 1890s, many orthodox Hindus claimed that indoor cremation was utterly alien to them and incompatible with their traditional funeral rites. As one European participant in these debates put it, seeking to pinpoint the distinction between two very different views of cremation, “The Hindoo motive for burning a body is to prevent defilement of the dead; the motive for the European is to prevent defilement of the living” (*Times of India*, 11 October 1887: 5). At best, “scientific” cremation was held by opponents to be fit only for those who died homeless and unclaimed on the streets (*Times of India*, 4 March 1887: 6; 19 August 1887: 3; 17 September 1887: 6). In this regard, municipal cremation bore something of the same stigma as the dissection of unclaimed paupers in nineteenth-century Britain, a maltreatment of the dead inflicted on those too poor and powerless to escape such a demeaning fate (Richardson 1988). If in Britain cremation progressed down the social scale from the upper and middle classes, in India Western-style cremation began with the low-caste poor and struggled to gain acceptance among high-status communities.

Colonial policy might further technological change, but not necessarily the specific forms of technology the West itself favoured. Thus, a further factor in the increasing recourse to cremation in India lay in changes in colonial administrative practice, especially in the jails. Until the 1830s it had been customary to bury the bodies of Hindu prisoners, unless they belonged to the higher castes, and the cost of their cremation was met by relatives or prisoners of their own caste.^9^ However, in response to demands from prisoners themselves, it became the norm to cremate all Hindus and Sikhs, unless relatives requested the return of their bodies. Even then a corpse would not be released for extramural cremation or burial if the authorities believed that it might be used in anti-government demonstrations. By the early twentieth century the practice of cremating the unclaimed bodies of Hindus and Sikhs who died in prison, had become formally incorporated into provincial jail regulations—just as Muslims and Christians were routinely buried.^10^ Similarly, outside South Asia, the dispersal of Indians throughout the British Empire and beyond led to requests from overseas Hindus and Sikhs that their co-religionists should be cremated according the requirements of their faith. For them the argument was religious, not sanitary. From places as far apart as Shanghai, Fiji, Gibraltar, Addis Ababa, British Honduras and the United States, the India Office and the Colonial Office in London encountered claims (which they sometimes spurned) for the recovery of cremation expenses for “pauper Indians”. They also faced demands from diasporic Hindus and Sikhs for the right to cremate their dead and have officially assigned space for that purpose.^11^ This pan-imperial, and increasingly transnational dispersal of open-air Indian cremation practices was not uncontested. In many places the civil authorities or local residents continued to regard cremation as uncivilized and un-Christian or, as in the Middle East, un-Islamic. A more technical objection was that cremation might conceal poisoning and other murderous acts since it destroyed almost all physical traces and precluded exhumation.^12^ This was one of the grounds on which opponents resisted cremation in Britain as well as in India (Robinson 1889: 47–54; Hehir 1913: 408), but by the 1930s advances in forensic science overcame this objection, making it technically possible to detect metals and poisons in bone fragments and human ashes (Ghosh 1930: 340).

Thus, while India in some respects provided a positive model for cremation, and functioned as a centre for its global diffusion, the Indian version of open-air cremation was very different from the “scientific” cremation being developed in the West. While in India cremation continued to be a highly visible process, with wood pyres and burning grounds open to the elements, in Western countries cremation was performed indoors, out of sight, in enclosed, purpose-built structures, using gas or electric burners that generated very high temperatures and so rapidly reduced a corpse to ashes. In India, where the earlier form of cremation continued largely unmodified, not only was the technology pre-industrial, it was also sanctioned by rite and custom. During the time it took for fire to consume the body a series of rituals had to be performed, including the chief mourner’s breaking the charred skull of the deceased (Rambachan 2003: 645). The visibility of the body during cremation and the continuing attendance of mourners and priests for most of the burning were aspects unmatched in Western cremation. Yet despite these fundamental differences, since the mid-nineteenth century indoor crematoria was being promoted/recommended in India too. In 1864 (and thus well before similar structures were being commissioned in Europe), Major Thomas Martin designed a five-chambered crematorium in Poona (Pune) in western India in that would incinerate up to ten bodies at the same time with the “poisonous” fumes from the burning chambers dispelled through a tall chimney. The separate chambers were intended to allow Hindus of different castes to be burned independently and for their ashes to be collected unmixed with (and unpolluted by) those of other castes (Martin 1864). In the 1860s a “cinerator” had allegedly been constructed in Calcutta but with no more than Hindu “acquiescence” (Eassie 1875: 97–98)—there is scant evidence for its actual use. Four decades later a Cremation Society was set up in Calcutta, mainly patronized by Europeans. A small crematorium was constructed, equipped with a gas furnace imported from Paris at a cost of 40,000 rupees. The latest cremation technology thus travelled to India but to little effect. Even though the charge for each cremation was modest, only five or six cremations were performed annually during its early years. Most Britons retired back to their own country and died there, while the largely Roman Catholic Eurasian and Indian Christian communities were opposed on religious grounds to burning their dead (*Times of India*, 5 June 1913: 8). Only gradually, and increasingly following India’s independence in 1947, crematoria began to gain acceptance among the more secular elements of India’s high-status communities.

Cremation was, and in some respects remains, a practice closely identified with India, though not always positively. Especially in Muslim countries it continues to be regarded as indicative of a backward and uncivilized society (Ghosh 1992: 125–126, 235). The destruction of human remains by fire was a widely practiced technology, which in the Imperial Age was widely disseminated across the modern world, in part due to the highly visible example India provided as well as the presence of cremation-practising Hindus and Sikhs in the South Asian diaspora. At the same time it is striking to which extent “scientific” cremation, as developed by the West, largely failed to displace the ancient, indigenous practice in India. Yet not for the first time “traditional” and “modern” technologies constantly intersected, complemented, and competed with each other in this regard. Scholars have noted how the creation of a railway system in India facilitated Hindu pilgrimage to traditional sites rather than, as suggested by a colonial “civilizing” agenda, rendering them obsolete (Ahuja 2004: 95–116). Likewise, modern modes of transport, including air-travel, now allow Indians all around the world to either carry the ashes of their deceased back to India for dispersal in the Ganges, or even to have their bodies flown to India for cremation. Modern technology has made the Indian form of open-air cremation more than ever visible, familiar and symbolic.

**Fig. 2:**
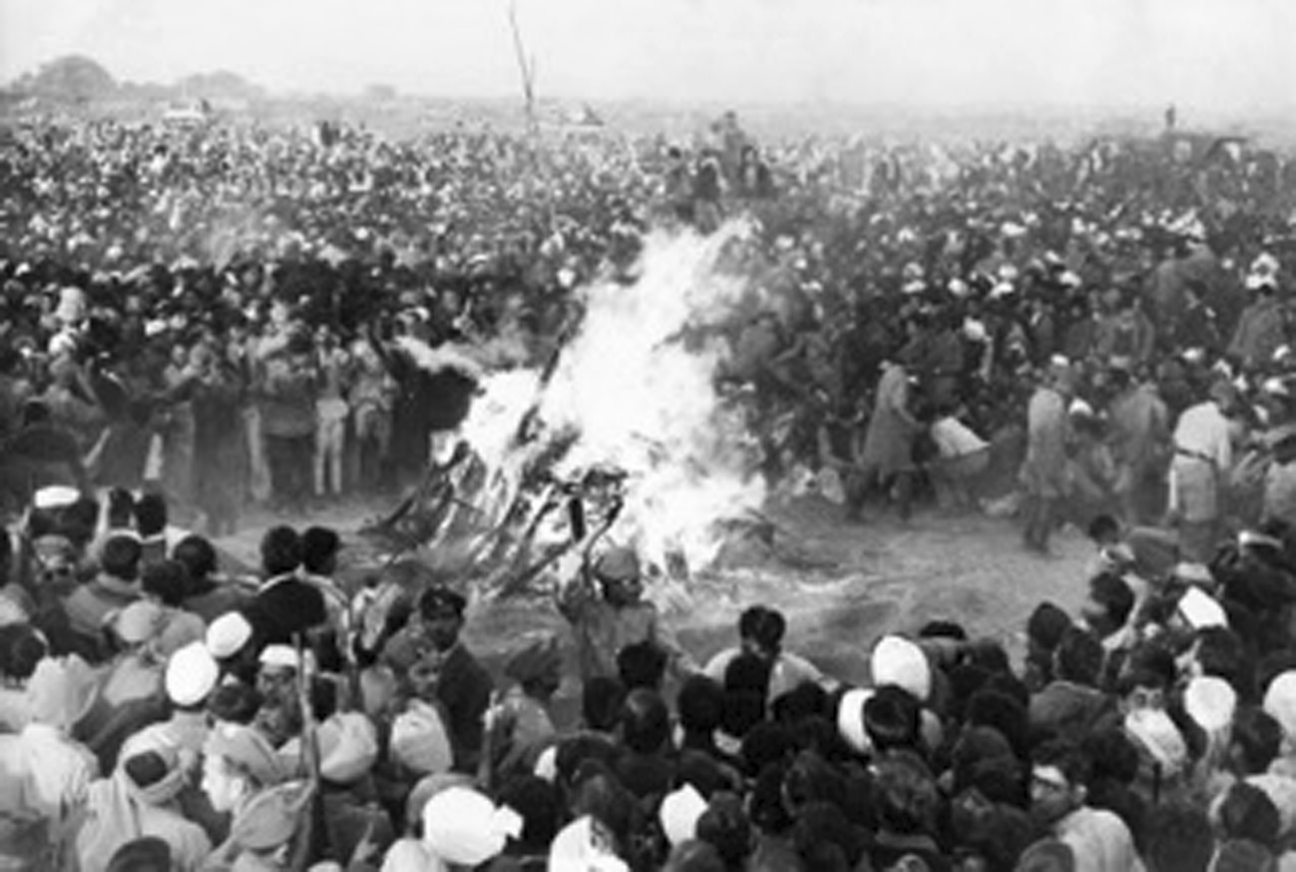
Cremation of Mohandas Gandhi, Delhi, January 1948

The Indian mode of cremation retained a cultural prestige and social value that the industrial-style crematorium of the West failed to usurp culturally and politically. As an essentially public act, cremation created political opportunities that worried the colonial authorities. This was especially the case, after the provincial government allowed the Hindu nationalist leader Bal Gangadhar Tilak to be cremated on Chowpatty Beach in Bombay in August 1920, that quickly became a memorial site and a focus for anti-government demonstrations (*Times of India*, 4 November 1933: 14). The very public (and internationally reported) cremation of Mohandas Gandhi after his assassination in January 1948, followed by that of Jawaharlal Nehru and other political leaders over the following decades, demonstrated not only to India but to the world at large the importance of cremation in the visualization of the Indian nation. Coverage and, even more so, photographic images of these ceremonial burnings were relayed nationally and internationally through newspapers and by means of radio and television.^13^ Since at least the 1920s, cremation has come to be regarded as emblematic of Hindu India and, by extension, the Indian nation. Denying Indians the right to cremate their dead became regarded as an insult to Indian nationhood (*Times of India*, 19 September 1929: 12). Cremation became a right, not just a rite. When Indians soldiers, airline pilots or politicians died overseas, it was a mark of national respect that they should be ritually cremated, and for this event to be publicized and memorialized in the national press.^14^ Raj Ghat on the banks of the Yamuna in Delhi, where Gandhi, Nehru and others leaders were cremated, has become a shrine to Indian nationalism. In the iconography of Indian nationhood, cremation has emerged as a symbol, emblematic (as *sati* once was) of suffering, sacrifice and devotion. Modern technology—air-travel, photography, mass media—has not dispelled Indian cremation practices, but rather given them a new visibility and an enhanced political valence. By contrast, cremation among those of South Asian ancestry in Britain has moved in the opposite direction. With the open-air burning of bodies prohibited by law and many of the practices traditionally associated with Indian cremation denied or curtailed, the burning of dead Hindus and Sikhs has had to conform, however reluctantly, to the “scientific” cremation of the West (Laungani 1996; Rambachan 2003: 645–646).

## Burning Waste

According to Ludovico Brunetti, one of the pioneers of crematoria technology in 1870s Italy, cremation was about “burning a special sort of rubbish: the human body” (quoted in Laqueur 2015: 499). Discussion of cremation leads us logically to wider questions of waste-disposal and to the environmental governance of Indian cities. That cremation and incineration were closely connected subjects is evident from the comments of many contemporaries. This was partly due to the fact that the industrial technologies developed in relation to one were pertinent to the other, but also because they both related to questions of environmental management—that is, effective waste-disposal and the regulation of smoke pollution—that ranged high on the agenda of the municipal authorities established in India between the 1860s and 1880s. In British India it was often the municipalities that provided technological agencies and infrastructures, or, conversely, and despite their limited financial resources and executive powers, sought to manage the impact of urban expansion, industrial growth and environmental change (Mann 2007; McFarlane 2008; Arnold 2013b). Exemplifying the linkage between cremation and incineration, an editorial in the *Indian Medical Gazette *in 1887 rapidly passed from advocating the burning of corpses on sanitary grounds to favouring, for similar reasons, the burning of Calcutta’s municipal waste (Editorial 1887: 175). In the sanitary textbooks and manuals of the period the disposal of corpses sat next to the discussion of urban waste disposal, the regulation of “dangerous trades” and environmental “nuisances” (Bengal 1887: 92). What happened in the countryside was of far less concern, but in crowded cities like Calcutta and Bombay, with populations exceeding half a million people by the 1870s and rising towards one million by the 1900s, the disposal of the dead and the destruction of rubbish were matters of similarly pressing concern.

However, burning rubbish carried none of the sanctified status and cultural prestige that was inherent to the open-air cremation of human remains. For Hindus the use of fire to destroy a human corpse was purifying and ennobling; its equivalent use to destroy urban waste was likely to be deemed offensive and polluting in a ritual as well as environmental sense. Where cremation traditionally served the funeral needs of the higher castes, waste disposal was identified with the very lowest strata of Hindu society, the Dalits also known as “Untouchables”. In Bombay’s primitive sanitary infrastructure “untouchable” workers collected human excrement from houses by what was all too eloquently styled the “hand removal system”. They also gathered up street waste with bullock-carts and transported it to municipal depots for disposal (Tulloch 1872: 12–17; James 1906; Clemensha 1910: 1–3). Like open-air cremation, the socio-political infrastructure of colonial India allowed a pre-existing technological practice (using the manual labour of traditional “sweeper” castes as a sanitary agency) to continue or even find a new physical space and mechanical function within the modern city. Lacking a more effective or extensive system of underground sewers until the 1870s, much of the collected street litter and human sewage was dumped into Bombay harbour or tipped into Calcutta’s River Hooghly (Harrison 1994: 202–226). When this proved objectionable on health grounds, urban waste and “night-soil” were transported to the outskirts of the cities and used to reclaim swamps and waste ground, despite the swarms of flies this attracted and the stench of decaying rubbish that drifted back over the city. By the mid-1870s, Bombay’s 2,500 municipal sweepers and rubbish collectors, with their 350 carts, collected 180 tons of human waste and 118 tons of garbage a day (Bombay 1876: 112–113). By 1900, as the volume of waste continued to grow, 1766 cartloads of refuse and sweepings, amounting to 883 tons of rubbish a day, were removed, mostly for landfill (Goodrich 1901: 253). In Calcutta, 900 carts removed between 1,000 and 1,500 tons of refuse a day from the city (Simpson 1908: 243).^15^ As with cremation, the mounting sense of crisis surrounding urban waste and sewage disposal was intensified by the catastrophic outbreak of plague in Bombay in 1896 and the conviction that “filth” contributed to its generation and transmission.

By 1900 new technological solutions were urgently needed to improve the sanitary infrastructure and waste-disposal in the “contaminated city” (McFarlane 2008). Light railways were built, or existing tracks utilized, to speed the removal of waste to outlying dumping grounds. However, in Bombay the resulting stench drew protests from rail passengers and operating companies alike and had to be stopped (Bombay 1905: 190–193; Bombay 1908: 135). The municipality introduced mechanical vehicles to collect and remove waste from the streets in a bid to make the process more efficient and less offensive to the public (Turner 1914, chap. 2). But one alternative to the costly and laborious transporting and dumping or burying of waste was to burn it. There was nothing new, in principle, about burning waste. Traditionally, urban debris, including leaves and paper, was burnt on the streets by residents and shopkeepers; but fresh efforts were made to systematically burn rubbish collected from the streets outside the city on its thinly populated margins. The municipal authorities hoped that this would be a cheaper and more efficient means of disposal. However, the incineration of urban waste created problems of its own. Like the smoke issuing from cremation grounds, burning rubbish generated large quantities of dark, pungent smoke that engulfed residential areas and drew irate protests from Europeans and Indians alike. In the 1870s members of the elite European Byculla Club complained about the “foul, dense smoke” from waste burned on the nearby Tardeo Flats. Since smoke pollution was one of many urban “nuisances” that the civic authorities were committed to curtailing, they could not ignore such influential complaints and the municipality was forced to abandon burning waste in the open (Arnold 2013b).

A second complication was that Indian street litter proved to be more voluminous and more difficult to burn than equivalent waste in Britain, due to the great quantity of vegetable matter and the small percentage of cinders it contained. According to Bombay’s health officer, London waste consisted of 64 percent cinders and ashes, but in India (where cow-dung was more common than coal as a domestic fuel) the equivalent waste contained less than two per cent ashes and cinders, but had large quantities of vegetable waste (including leaves used as plates) and so possessed little calorific value. In terms of volume, too, the residents of Bombay produced on average 0.36 tons of waste a head per year, substantially more than the 0.25 tons of English cities (Turner 1914: 120–125). Indian waste was, therefore, more difficult—and more costly—to burn. Despite this, and concerned about high transport and disposal costs, India’s municipal authorities began to explore other technological solutions. From the late 1880s on they experimented with modern incinerators (“destructors”), seeking to apply machines and methods first developed for waste-disposal in Britain’s industrial cities to very different Indian conditions (Saunders 1881). In 1889, the *Indian Medical Gazette *welcomed trials in burning Calcutta’s waste in incinerators rather than dumping it in the nearby salt lakes. Indeed, the *Gazette* looked forward to the time when “every town and townlet, hospital and prison” would have its own incinerator “not only for garbage, but also for all forms of refuse” (Editorial 1889: 275). But six years later the *Gazette* was less enthusiastic, citing as objections the smell and smoke produced by incinerating wet, bulky waste and the high cost involved (Editorial 1894b: 381). If the purpose of incineration was to use the combustible properties of the waste itself, aided by only a minimal quantity of added fuel, to consume garbage, then in India’s cities this principle seemed hard to realize in practice. As with open-air cremation, disposing of municipal waste by means of fire seemed an inadequate means of destruction.

Bombay, too, experimented with a number of “crude incinerators” before the municipal engineer, Muncherji Cowasji Murzban, decided to install a more sophisticated British destructor. But the trial met with only moderate success and was eventually abandoned, again because of the resulting “smoke nuisance” and the stench of piled-up garbage awaiting incineration. Due to the high vegetable content of street rubbish in India, the incinerator generated large quantities of black smoke, and during the monsoon season needed additional (and costly) fuel to reduce the damp and voluminous waste to ashes (Bombay 1894: 466–467; Bombay 1895: 523). The city then reverted to using urban waste to reclaim low-lying land on the city margins, land that in the short term could be used for growing crops and later for building purposes, thus securing a substantial financial return for the municipality. Perhaps because of its ritually polluting associations, relatively little of urban India’s waste and sewage was used as manure for agricultural purposes. Besides, the continuing availability of low-cost Dalit labour to collect, spread and bury the rubbish made this a relatively cheap, if low-tech, option compared to the expense of buying, installing and maintaining a destructor. To a zealot like Francis Goodrich (1901: 253–254) abandoning incineration in Bombay was a “very extraordinary” decision to take in the light of “modern British practice”. But even though colonial India instinctively looked to Britain for technical expertise to improve sanitation and curb smoke pollution (Grover 1903; Nicholson 1906), metropolitan advice was not always heeded. The “rule of experts” (Mitchell 2002), especially when they had no prior knowledge of Indian conditions, often counted for less than the perceived importance of local environmental, social and economic factors. Bombay’s seemingly retrograde step in abandoning destructors might run counter to British expertise, but locally it appeared to make sense.

With a population of approximately half a million in 1911, India’s third city, Madras (Chennai) presented a further example of the reversal in waste incineration policy. By 1913 the municipality had installed two large destructors to dispose of its 324,202 cartloads of rubbish a year as well as 36 small incinerators for individual wards (Madras 1914: 16–17). However, within five years the municipality had abandoned the big incinerators except to burn the thousands of stray dogs and rats killed to curb rabies and plague, and had reverted to dumping in landfill sites. The inefficient working of large incinerators and the greater commercial value gained by reclaiming wasteland combined with belief in the sanitary benefits of infilling marshy ground and eliminating malarial mosquitoes (Madras 1916: 17; Madras 1920: 30–31).

India was not entirely alone in its difficulties. Destructors encountered many problems in Britain between the 1870s and 1900s, problems that took decades to resolve. Although their export from Britain to other parts of the Western world provides in some respects a case-study in technology diffusion (Melosi 1988) (as the example of the United States showed) there were numerous technological, environmental and social reasons why urban incinerators enjoyed only fitful support from municipal authorities and the general public. In many American cities dumping remained a preferred (and less costly) alternative (Melosi 2000: 196–203, 275–278, 347–349). However, many of the problems experienced in India were interpreted as being specific to that country or to sanitary practice and waste-disposal in “the East” more generally. A substantial volume of literature between the 1880s and 1920s emphasized the exceptional impediments to the removal and destruction of rubbish in the tropics, and so further validated the problem of “the tropics” as understood in contemporary Western science, medicine and environmental thought (Arnold 1996: 141–168). This was an argument grounded in perceived physical differences between West and East, such as India’s hot, moist, monsoon climate, but it also encompassed social and cultural practices, such as religious beliefs and even the manner in which Indians chose to dispose of the human dead as well as urban waste (James 1906; Williams 1924). A former Calcutta health officer summed up this position when he wrote that “The hygiene of the Tropics and of warm climates is the same in principle as the hygiene of colder climates.” But he then added: “the differences of temperature, food, mode of life, environment, and civilization to be found in the Tropics modify to a considerable extent the conditions under which those principles have to be applied”. In consequence, “the practice of hygiene in the Tropics differs in many respects from that pursued in colder climates, and that which is suitable to the latter is not always suitable to the former” (Simpson 1908: v). Expectation as much as experience fuelled the sanitarian conviction that Western solutions to the problem of municipal waste (any more than to those posed by cremation) were not necessarily practical, or even desirable, in the East.

## Incineration and Innovation

Taking the view that “large destructors seldom work satisfactorily in India”^16^ governments and municipalities across the country sought a solution to the urban waste problem by amending the design, scale and function of incinerators in order to make them more compliant with local conditions. Thus alongside large incinerators, mostly of British manufacture, trials were conducted with smaller, cheaper, locally made machines.

Around 1910 C. L. T. Griffith, chief engineer to the Madras Corporation, designed and tested a small incinerator of his own. Unlike the imported iron monsters, his incinerator consisted of a simple brick structure, with a chimney 12 to 25 feet high, which cost no more than 125 rupees to construct. But significantly Griffith substituted “traditional” labour for “modern” know-how, for its functioning depended on the availability of a low-paid, local workforce. The preliminary sorting of the waste delivered to depots was done, not by mechanical conveyor-belts and sieves of various gauges, but by men armed only with rakes and forks, and by women and children using their bare hands to separate combustible from non-combustible material. As the process included the extraction of human excrement from domestic waste and street rubbish, this was the kind of unhealthy and degrading work only “untouchables” were prepared to undertake. Even so, Griffith’s small incinerator was hailed as “a sanitary and financial success” and his experiment encouraged other local initiatives to develop small incinerators that were more practicable than the giant, multi-oven destructors (Turner 1914: 160–162). However, when the trials in Madras were extended to include burning excrement (collected from nearby Dalit colonies) they soon had to be abandoned. “The gases given off from the incinerating night-soil”, reported the city health officer, “proved such a vile nuisance, that I was compelled to discontinue these experiments” (cited in Turner 1914: 162).

Although initially intended to address a problem of municipal waste management, the move early in the twentieth century towards locally designed, small-scale incinerators proved successful in other locales too. Conspicuous among these were army cantonments, where protecting soldiers from cholera and typhoid was a paramount concern and where issues of cost were less restrictive than in cash-strapped municipalities. In the 1890s a series of experiments began in northern India using small incinerators to burn general waste but also to destroy human excrement (in preference to the older “trenching system” in which faeces were buried in the soil). Since several small incinerators could be located at different points around the cantonment and excreta could be thrown directly into the furnace, this technology promised to do away with the need for foul-smelling conservancy carts (Haines 1907: 204–206; Hamilton 1907: 151–152; Morris 1907: 374–375; Young 1907: 331–333). Even small incinerators could generate relatively high temperatures and use different kinds of dry refuse as fuel. However, the quantities of waste that could be consumed were small, the monsoon climate restricted their use during the rainy months when refuse was too damp to burn, and, to be efficient, they needed careful maintenance (Hehir 1913: 342–347). But, encouraged by the success of the cantonment trials, experimental incinerators were designed and built for use in urban residential compounds.^17^ Thus incineration technology moved closer to being of “everyday” utility. Made from recycled bricks and scrap metal, with a few iron bars to separate the fuel from the waste matter, these were easily erected alongside household latrines. The cost involved was minimal and in theory they allowed both domestic waste as well as human excrement to be destroyed onsite rather than being transported, at greater cost and inconvenience, to a distant municipal depot. Little by way of smoke or smell was reportedly produced (some reports suggested otherwise) and the small residue of ash could readily be disposed of, perhaps in the flowerbeds. But again the incinerator relied on the customary labour of the household sweeper, or *mehter*, to feed the incinerator with faeces and waste and to keep it burning long enough and with sufficient intensity to destroy all the debris fed into it. While conventionally trusting the participation of the “wily *mehter*” (Haines 1907: 205), these experimental incinerators also demonstrated the value of a new technological aid—kerosene. Wood-shavings, waste paper and dry leaves were seldom enough to get a small incinerator going or keep it alight. Kerosene, from the 1880s widely sold on Indian streets and in bazaars, was added to sanitize and deodorize the material fed into the furnace and to make combustion more rapid and intense (Editorial 1894b: 382; Young 1907: 333).

Small destructors became commercially widely available in India and were particularly recommended for institutions—for destroying the ejecta and excreta of hospital patients or in prisons where their use could be closely monitored (Simpson 1908: 336–337; Ghosh 1930: 267–268). Yet, for all this flurry of innovation, the small incinerator in India had little long-term impact, perhaps because householders lacked sustained enthusiasm for these potentially smelly and smoky devices, situated so close to kitchens and bedrooms, or because Indian sweepers took little interest in their upkeep. The 1923 edition of *McNally’s Sanitary Handbook* discussed incinerators, large and small, at length before arriving at a less than favourable conclusion. Large destructors, it reported, were expensive to operate and struggled, even in Europe, to burn all the waste fed into them: in India this was “a still greater source of trouble”. The machinery required “constant care and attention from an intelligent workman”—implying that few such operatives existed in India—and so, “generally speaking, these destructors are unsuitable for India” (Russell 1923: 146–147). Small incinerators fared little better, especially when tasked with the destruction of human faeces. “Very careful supervision is required”, *McNally’s* warned, “as not only is the mixing platform a danger to health, but the gases from the half-burned night-soil have an unbearable odour.” Incinerator technology as a whole was deemed “expensive and wasteful” and only fit for hospitals and jails “where it is necessary to destroy completely the excreta in the case of intestinal diseases like typhoid, cholera and dysentery, and where those employed in carrying out this work can be carefully supervised” (Russell 1923: 152). Among the various sewage-(rather than waste-)disposal technologies available to India around 1900–1910, septic tanks enjoyed greater favour among public health experts: some even preferred a return to the old “trenching” system (Simpson 1908: 229–233; Clemensha 1910: 33).

Instead of wholeheartedly adopting Western incinerator technology, India fell back on an unsatisfactory (and often unsavoury) mixture of old and new waste-disposal technologies, often glancing, with apparent envy, at the brave new world of urban incinerators in the United States and Europe (*Times of India*, 19 November 1953: 8). As late as 1949, the municipal authorities in Bombay were still struggling to introduce a pilot scheme to incinerate the 2000 t of rubbish by then being produced every day in the city and to end the dumping of waste in marshes and creeks (*Times of India*, 27 January 1949: 7). Apart from the growing size of the urban rubbish mountain, little had seemingly changed since the 1910s. Nor did the problem of the apparent incompatibility of Indian waste with modern technology evaporate with the passing of empire. An ultra-modern incinerator, costing ten million dollars, installed in Delhi in 1990 and funded by the Danish aid organization DANAID, ceased operating within a week because the waste proved unsuitable. As one commentator observed, “with a low consumption society and a very high rate of scavenging and recycling, what garbage is left in India is too wet to burn” (Puckett 1998). This was not quite a complete revisiting of the waste-disposal difficulties identified a century earlier, but the episode is suggestive of the way in which a long-standing sense of difference between India and the West still remained.

## Conclusion

Over the past thirty years there has been a marked shift away from a unilineal model of imperial agency and technological diffusion from Europe to its colonies, to a multi-directional and multi-sited history of networking, hybridity, connectedness and mutuality (Hodge 2011: 3–29). This discussion of cremation and incineration in nineteenth- and twentieth-century India has sought to highlight some of the complexities involved in the adoption and deployment of technologies using fire as a modern means of destruction—whether of human corpses or urban waste. These were issues that have come to assume a global importance in the modern age: one must consider how cremation has progressed from illegality and obscurity in Britain in the 1870s to being the preferred means of disposing the dead among more than 70 percent of the British population in the 2010s, and how cities around the world have struggled to find suitable means to destroy or recycle ever-growing mountains of waste. These, then, are not obscure technologies, but they have been deployed over the past 150 years in ways that deny a simple diffusionist narrative and emphasize the importance of the physical environment and social context, the interplay of resistance, cultural choice and selective appropriation. Technologies travel in various ways and not exclusively from European “core” to colonial “periphery”. Technology, hailed in a more imperially minded age, as the exemplar of Western power and ingenuity, often in practice revealed its vulnerability, its humbling reliance upon local mediation and “provincial” agency.

In the West, where cremation brought industrial design to the demolition of the dead, India still served as a model of sorts, even though its pre-industrial mode of open-air cremation was often deemed morally dubious and technically defective. Conversely, when Western cremation technological travelled, as both idea and practice, to India, it gained little traction, given the attachment of those who already followed cremation there to a rite and a process that owed nothing to Europe. Growing public and scientific support for cremation in the West did, however, help create the institutional and social space for the older form of cremation to be retained, incorporated into the modern cityscape and internationalized. Rather than becoming obsolete and archaic, open-air cremation became for many Indians emblematic of their modern faith and national identity. Where “scientific” cremation was adopted, it was often not a prestigious rite but demeaning in relation to the unclaimed poor. The incineration of urban waste held none of cremation’s socially positive connotations, but it, too, acquired prominence as a Western technological solution to an Indian problem of urban waste-disposal. Though it garnered some success as a form of technology transfer, it too failed in supplanting other forms of waste-disposal in India, partly due to environmental complications (smoke pollution, incombustible waste, the need for urban landfill), but also owing to a pre-existing social infrastructure of “untouchable” sweepers, who continued to retained a vital role in urban sewage- and waste-disposal. Technology might travel, but in the process it often underwent a radical transformation or was obliged to operate symbiotically with other, older technological forms and practices.

## Footnotes

1 For an illustration of the importance of the smoke pollution issue in Indian cities and attempts to control it, see Anderson (1995). There is an extensive scholarly literature on India’s urban waste in general, notably Chakrabarty (1991) and Kaviraj (1997); see also Mann (2007); Arnold (2013b); Sharan (2014).

2 India Office Records (hereafter IOR), British Library London, Bengal Judicial (Jails), nos. 2–4, 11 August 1859.

3 IOR, L/PJ/6/743: 83.

4 This would need to set against the far greater consumption of timber and forest destruction due, for instance, to the fuel needs of the Indian railways in the late nineteenth century (Das 2013).

5 Cremation at Benares (Varanasi) and the immersion of ashes from cremations elsewhere have been a significant source of pollution in the Ganges. In recent years as many as 32,000 corpses a year have been burnt at the *ghats* there with more than 300 tons of half-burnt bone and flesh emptied into the river: Rowlatt (2016).

6 On Woking crematorium, see Laqueur (2015: 517). Cremation only gradually gained popularity in Britain, rising from very small numbers in the 1880s to over 70 percent by 2000 (Parsons 2003: 612–613).

7 In Madras, too, burial rather than cremation was the more common means of disposing of the Hindu dead. In 1914, 5,362 bodies were cremated (all Hindus) compared to 18,812 buried, including 13,919 Hindus (Madras 1915: 59–60).

8 In 1920 a resolution passed by the Mahar Parishad called on members of the Mahar “untouchable” community to cremate rather than bury their dead (Gokhale 1993: 69).

9 IOR, Bombay Judicial, April 1836.

10 IOR, L/PJ/7/476.

11 IOR, L/PJ/6/743: 83; L/PJ/6/1241: 1830; L/PS/11/281; L/PJ/6/1249: 2339.

12 As in Fiji: IOR, L/PJ/7/476.

13 For Gandhi’s cremation, see *Times of India*, 4 February 1948: 5. On the international coverage of this event, see Cookman (1998). Indira Gandhi’s cremation in 1984 was broadcast live on BBC television.

14 As with the accidental death of two Indian soldiers in the Gaza strip in 1960 (*Times of India*, 19 January 1960: 7).

15 Summary accounts of sewage and waste disposal in Calcutta are given in Headrick (1988: 152–159) and Harrison (1994: 202–226).

16 IOR, Madras, Local and Municipal, Government Order 1678, 1 September 1918.

17 Small incinerators also found favour in other tropical colonies, including in West Africa: see Muirhead (1914).
